# Post-tonsillectomy outcomes in children with mucopolysaccharidosis and obstructive sleep apnea

**DOI:** 10.1186/s40463-023-00685-y

**Published:** 2023-12-24

**Authors:** Zachary Elwell, David Mancuso, Nikolaus E. Wolter, Evan J. Propst, Tulio Valdez, Patrick Scheffler

**Affiliations:** 1 Department of Otolaryngology – Head and Neck Surgery, University of Arizona College of Medicine – Tucson, 1501 N Campbell Ave, Tucson, AZ 85724 USA; 2 School of Molecular Sciences, Arizona State University, Tempe, AZ USA; 3 Department of Otolaryngology – Head and Neck Surgery, Hospital for Sick Children, Toronto, ON Canada; 4 Department of Otolaryngology – Head and Neck Surgery, Stanford University, Stanford, CA USA; 5 Division of Otolaryngology – Head and Neck Surgery, Phoenix Children’s Hospital, Phoenix, AZ USA; 6 Department of Child Health, University of Arizona – Phoenix College of Medicine, Phoenix, AZ USA; 7 Department of Surgery, Creighton University School of Medicine, Phoenix, AZ USA

**Keywords:** Mucopolysaccharidosis, Adenotonsillectomy, Postoperative complications

## Abstract

**Objective:**

To describe the incidence of respiratory complications, postoperative hemorrhage, length of stay, and cost of care in children with mucopolysaccharidosis (MPS) undergoing adenotonsillectomy (AT).

**Methods:**

Analysis of the 2009, 2012, and 2016 editions of the Healthcare Cost and Utilization Project Kids’ Inpatient Database (HCUP KID) identified 24,700 children who underwent AT (40 children with MPS). Demographics, respiratory complications, postoperative hemorrhage, length of stay, and total cost were compared across children with and without MPS.

**Results:**

Children with MPS had a higher likelihood of being male (*P* < 0.017). There was a higher rate of respiratory complications in children with MPS compared with children without MPS [6/40 (15%) vs. 586/24,660 (2.4%), *P* < 0.001], which remained significant after adjusting for sex [adjusted odds ratio 6.88 (95% CI 2.87–16.46)]. There was also a higher risk of postoperative hemorrhage [4/40 (10%) vs. 444/24,660 (1.8%), *P* < 0.001), with sex-adjusted odds ratio of 5.97 (95% CI 2.12–16.86). Median (IQR) length of stay was increased in children with MPS (3 days, 1–4) compared with children without MPS (1 day, 1–2, *P* < 0.001). There was an increase in median (IQR) charges for hospital stay in children with MPS compared with their peers [$33,016 ($23,208.50–$72,280.50 vs. $15,383 ($9937–$24,462), *P* < 0.001].

**Conclusions:**

Children with MPS undergoing AT had an increased risk of respiratory complications, postoperative hemorrhage, longer length of stay, and a higher cost of treatment when compared with children without MPS. This information may help inform interventional, perioperative, and postoperative decision making.

## Introduction

Pediatric obstructive sleep apnea (OSA) is among the most common obstructive airway diseases in the pediatric population. The American Academy of Pediatrics (AAP) states that the current prevalence rate of OSA based on level I and II studies ranges from 1.2 to 5.7% in the general population [1]. The prevalence of OSA is significantly higher in patients with underlying neuromuscular conditions and craniofacial anomalies. A study conducted by Gönüldaş et. al. showed a significantly higher incidence of OSA in children with mucopolysaccharidosis (MPS) tested with polysomnography when compared to the general pediatric population [2]. If left untreated, OSA can lead to the development of adverse developmental and health outcomes including learning disabilities, delayed physical growth, disruptive behavior, hyperactivity, adverse metabolic functioning, and cardiovascular complications including hypertension [3–14].

MPS refers to a group of inherited metabolic disorders characterized by the deficiency of enzymes required for the breakdown of glycosaminoglycans (GAGs), previously called mucopolysaccharides. The dysfunction or absence of one of these enzymes prevents the degradation of GAGs, resulting in the accumulation of partially degraded GAGs in lysosomes, ultimately causing cellular dysfunction and specific clinical abnormalities [15]. There are seven distinct subtypes of MPS that vary widely based on the affected enzyme and metabolic pathway, resulting in these clinical presentations [16–22].

The palatine tonsils and the adenoid glands are the most common areas of upper airway obstruction in pediatric patients with OSA [23]. The AAP recognizes adenotonsillectomy (AT) as the first line treatment for pediatric OSA [24–27]. A combination of adenotonsillar hypertrophy, craniofacial anomalies, low muscle tone, and, in some cases, neurologic abnormalities predispose children with MPS to developing OSA. These factors may also impact the safety and effectiveness of AT in children with MPS. The objective of this study is to describe the incidence of respiratory complications, postoperative hemorrhage, length of stay, and cost of care in children with MPS undergoing AT.

## Materials and methods

### Data source

The 2016 Healthcare Cost and Utilization Project Kids’ Inpatient Database (HCUP KID) is the largest validated, publicly available all-payer pediatric inpatient care database in the United States. It includes 80% of pediatric (age < 21 years old) discharges from 4200 community hospitals in the United States. The HCUP KID has been produced every 3 years (1997, 2000, 2003, 2006, 2009, 2012), with a delayed release of 2015 data until 2016 because of the complexities of analyzing a mixed data year (2015 contained both ICD-9 and ICD-10 data). The 2016 HCUP KID is composed of ICD-10-CM/PCS data only [28]. This study did not require approval from the office of research integrity at Phoenix Children’s Hospital, as the HCUP KID is a publicly accessible database with de-identified data and is compliant with the Health Insurance Portability and Accountability act.

### Outcomes

Separate analyses were performed for race, age, sex, length of stay (reported in days), total charges (U.S. Dollars), and postoperative complications, including post-tonsillectomy hemorrhage and respiratory complications.

### Statistical analysis

All analyses were performed with IBM SPSS Statistics Version 29 (SPSS, Inc, an IBM Company, Chicago, Illinois). Comparisons of patient characteristics were performed using a χ^2^ test or Fisher exact test for categorical variables and a two-tailed independent samples t-test was performed to compare continuous variables. A binary logistic regression model was used to compare odds for postoperative respiratory complications as well as postoperative hemorrhage between patients with and without MPS. Given the difference in sex distribution between groups, sex was introduced as a covariate into the model, but not found to have a significant effect in either regression. Odds ratios were calculated with 95% confidence intervals (95% CI).

## Results

### Demographics

Table 1 illustrates that children with and without MPS each had a median age (IQR) of 3 (2–7) years. The majority of MPS patients were male, and this was significantly different from the control group (77.5% vs. 58.5%, *P* = 0.017).

**Table 1:** Patient demographics

		No MPS (N = 23,302)	MPS (N = 40)	*P* value
Race	Missing	1675 (7.2%)	3 (7.5%)	NS
	White	9868 (14%)	14 (35%)	NS
	Black	4802 (20.6%)	9 (22.5%)	NS
	Hispanic	4857 (20.8%)	9 (22.5%)	NS
	Asian or Pacific Islander	598 (2.6%)	2 (5%)	NS
	Native American	159 (0.7%)	0 (0%)	NS
	Other	1343 (5.8%)	0 (0%)	NS
Sex	Male	14,353 (58.5%)	31 (77.5%)	0.017
	Female	10,164 (41.5)	9 (22.5%)	NS
Median age (Years) (IQR)		3 (2–7)	3 (2–7)	NS

*MPS* mucopolysaccharidosis, *NS* not significant

### Hospital course: length of stay and total charges

Table 2 illustrates that median (IQR) length of stay was increased in children with MPS (3 days, 1–4) compared with children without MPS (1 day, 1–2, *P* < 0.001). There was an increase in median (IQR) charges for hospital stay in children with MPS compared with their peers [$33,016 ($23,208.50–$72,280.50 vs. $15,383 ($9937–$24,462), *P* < 0.001].

**Table 2:** Study outcomes in children with and without Mucopolysaccharidosis

Outcome	MPS	No MPS	P
Length of stay (days, IQR)	3 (1–4)	1 (1–2)	< 0.001
Total Cost (USD, IQR)	33,016 (23,208–72,280)	15,383 (9937–24,462)	< 0.001
Rate of PTH	15%	2.4%	< 0.001
Rate of Respiratory Complications	10%	1.8%	< 0.001

*MPS* mucopolysaccharidosis, *IQR* interquartile range, *USD* U.S. Dollars, *NS* not significant, *PTH* post-tonsillectomy hemorrhage

### Post-operative complications: post-tonsillectomy hemorrhage and respiratory complications

Table 2 illustrates that there was a higher rate of respiratory complications in children with MPS compared with children without MPS [6/40 (15%) vs. 586/24,660 (2.4%), *P* < 0.001]. In binary logistic regression, with sex introduced as covariate, having MPS significantly predicted the outcome of respiratory complications (*P* < 0.001), with an odds ratio of 6.88 (95% CI 2.87–16.46). There was also a higher risk of postoperative hemorrhage [4/40 (10%) vs. 444/24,660 (1.8%), *P* < 0.001) among patients with MPS. In binary logistic regression, with sex introduced as covariate, having MPS significantly predicted the outcome of postoperative hemorrhage (*P* < 0.001), with an odds ratio of 5.97 (95% CI 2.12–16.86).

## Discussion

This study analyzed outcomes of patients with MPS who underwent AT. Patients with MPS tended to be younger, have a longer length of stay, and have a higher total cost of hospital stay than patients without MPS.

We also found a significantly increased length of hospitalization and cost of care in patients with MPS, which is congruent with findings from previous studies for other procedures. More specifically, a total cost-analysis study by Davari et al. [29] recorded that the average annual cost of treatment for MPS-I patients is $87,971.99, a quantity 16.2 times the GDP per capita in Iran. A separate cost-analysis study by Conner et al. [30] recorded an average cost per patient per month of $10,715 for an average annual cost of $128,580 for medical care of patients with severe MPS-I. It is important to note that neither of these studies specifically investigated surgery-specific costs of care. The study by Davari et al. includes surgical costs in its estimate of the annual cost of care for MPS-I patients but does not provide a specific annual estimate of surgical care per patient. The study by Conner et al. only recorded costs for medical interventions and indicated surgical costs as an area for future study. Given the paucity of information in the available literature, quantifying the cost of surgical management of MPS remains a viable area for future study. However, when comparing MPS to other complex medical conditions, the general trend in the literature, which consistently demonstrates higher costs of care for children with complex medical conditions and congenital syndromes, applies directly to children with MPS both in terms of medical management as evidenced by the above studies, and from a surgical perspective as reported in our retrospective review of the HCUP KID database for patients with MPS undergoing AT.

Interestingly, there was a statistically significant trend toward an increased risk of post-tonsillectomy hemorrhage (PTH) in patients with MPS, with an adjusted odds ratio of 5.97. Increased post-tonsillectomy hemorrhage rates have been documented in other complex pediatric patient populations, such as patients with Down syndrome and cerebral palsy, but this has not been previously reported in MPS. Data on postoperative bleeding in patients with MPS across surgical disciplines is scarce. A PubMed review yielded a lack of studies that have specifically documented the incidence of postoperative hemorrhage in patients with MPS or the association between specific surgical procedures and postoperative hemorrhage in patients with MPS. Furthermore, in patients with certain subtypes of MPS, including MPS-IH (Hurler Syndrome), studies have shown coagulopathy directly related to glycosaminoglycan levels in the blood [31]. Whereas postoperative blood loss in patients with MPS undergoing AT has not previously been studied, this finding was statistically significant in our study. Thus, it is worth being aware of this potentially increased risk in this vulnerable patient population, and providers performing AT on patients with MPS should do so at tertiary care pediatric centers best equipped to manage these potential complications.

There was a significantly higher rate of respiratory complications in children with MPS compared to children without MPS, with an adjusted odds ratio of 6.88. To our knowledge, this finding has not been previously investigated. However, similar postoperative respiratory complications have been found in other children with congenitally complex airways. For example, a recent systematic review by End et al. investigated the risks and benefits of AT in children with cerebral palsy (CP) and OSA. They reported children with CP have a significant risk of developing postoperative respiratory complications, including pneumonia, and that these patients required additional airway management with increased requirement of unplanned ICU admission [32]. This pattern is consistent with our findings, which demonstrate a higher risk of postoperative respiratory complications in children with MPS compared to those without MPS.

To our knowledge, this is the first large database study examining post-tonsillectomy complications in this patient population. However, providers should note several limitations to the current study. The first limitation is the study’s retrospective database design. Further, our comparison group did not only consist of otherwise healthy patients but included all other pediatric patients undergoing AT. This design may have overestimated complication rates, length of stay, and costs in this group. However, our data still achieved statistical significance in several outcomes. MPS contains seven different unique subtypes. The HCUP KID database is based on ICD-10-CM/PCS data only. Therefore, there is a lack of granularity inherent to this database that, unfortunately, did not allow us to account for specific MPS subtypes or disease severity by creating a severity of illness subclass within those particular subtypes.

Furthermore, we could not differentiate between specific subtypes of respiratory complications or between primary versus secondary PTH. Since tonsillectomy status and the procedure code for PTH were part of the admission criteria, providers may assume that all instances of hemorrhage in post-tonsillectomy patients included in the HCUP KID database are primary hemorrhages. Similarly, polysomnographic data or information on OSA severity was not available. Patients with more severe OSA are at higher risk for postoperative complications, and due to the lack of this data, we were unfortunately not able to control for this confounder. There may be a difference in OSA severity between patients with MPS and other patients studied herein. However, given the nature of the HCUP KID database, we do not know this to be true without a doubt. However, the data collected from the HCUP KID database is all inpatient data. Therefore, all patients included and compared in the study were hospital inpatients. Apart from age, the most common reason for postoperative admission would be due to severe OSA or other comorbidities, such as congenital syndromes that predispose patients to severe OSA.

Furthermore, among children with severe OSA, there is no substantial evidence to support that increasing AHI is correlated with an increased risk of PTH. Therefore, the striking difference between patients with MPS and patients without MPS in our study is unlikely to be solely due to differences in OSA severity. In addition to disease severity, the HCUP KID database did not allow for granular analysis of additional potential confounders known to be associated with a higher risk of PTH, including but not limited to infection, hematologic diseases, and neurodevelopmental diagnoses. While our patients did not have additional hematologic diseases that are not inherent to MPS, the effects of MPS on the hematologic system are well-known and established. The inherent hematologic dyscrasias of MPS may be a confounder that was not adjusted for when considering PTH in patients with MPS. However, this increased risk is inherent to all patients with MPS.

Additionally, patients with active infections or poor functional status, on average, have been found to have increased rates of post-tonsillectomy complications compared to other patient populations, such as patients with cerebral palsy [33]. We did not have this data available through the HCUP KID database to address all confounders fully. Lastly, the proportion of male-to-female children was higher in the MPS group. There is evidence of an increased risk of PTH in male patients in the literature, and this could have been a confounding factor. However, our odds ratio was adjusted for sex and continued to demonstrate the robustness of the results of the current study.

## Conclusions

In summary, children with MPS undergoing AT had a longer length of hospital admission, higher cost of treatment, increased risk of postoperative respiratory complications, and increased risk of postoperative hemorrhage compared to children without MPS. This is important information that may help in consenting patients for AT and booking postoperative admissions. Additionally, this information is helpful to providers planning to perform AT on patients with MPS, which should be done at tertiary care pediatric centers well-equipped to manage these potential complications. This is the first large-scale database study investigating increased post-AT morbidity, specifically in the pediatric MPS population. However, limitations inherent to retrospective database studies do apply, and further prospective studies are needed to investigate AT in patients with MPS in terms of perioperative and polysomnographic outcomes.

## Data Availability

The datasets used and/or analyzed during the current study are available from the corresponding author on reasonable request.

## Abbreviations

MPS: Mucopolysaccharidosis
AT: Adenotonsillectomy
IQR: Interquartile range
OSA: Obstructive sleep apnea
GAGs: Glycosaminoglycans
HCUP KID: Healthcare Cost and Utilization Project Kids’ Inpatient Database
CP: Cerebral palsy
PTH: Post-tonsillectomy hemorrhage
NS: Not significant
USD: U.S. Dollars
